# Morphine and Angiogenesis in Hypertensive Animals

**Published:** 2012

**Authors:** Majid Khazaei

**Affiliations:** 1 *Department of Physiology, School of Medicine, Isfahan University of Medical Sciences, Isfahan, Iran*

In a recent published paper, Pourshanazari* et al* found that morphine enhanced angiogenesis in murine matrigel method in sham and two-kidney one-clip (2K-1C) hypertensive animals and they concluded that this effect is via NO pathway (1).

Morphine acts both directly and indirectly on angiogenesis, however, the results are contradictory. It seems that the different effect of morphine on angiogenesis process depends on the low or high dose and acute or chronic morphine administration. It is indicated that chronic morphine treatment in wound repair resulted in a significant decrease in the expression of HIF-1α and reduced vascular endothelial growth factor (VEGF) synthesis (2). Morphine increases systemic oxidative stress, impairs mobilization of endothelial progenitor cells (3) and inhibits hypoxia-induced VEGF expression in endothelial cells and cardiac myocytes in ischemic myocardium (4). Morphine also suppresses tumor angiogenesis through a HIF-1alpha/p38MAPK pathway (). In contrast, it is able to stimulate vascular endothelial cell proliferation *in vitro* through mitogen-activated protein kinase (MAPK) pathway (6). Topical use of opioids accelerate healing by up-regulating both endothelial and inducible nitric oxide synthase and the vascular endothelial-derived growth factor receptor Flk1 in the wounds (7). Thus, it seems that the effect of morphine on angiogenesis depends on the type of experimental model as well.

The authors also concluded that morphine enhanced angiogenesis in sham and 2K-1C animals via NO pathway and they showed that angiogenesis in hypertensive animals is much higher than sham group. Hypertensive subjects have reduced NO bioavailability due to several mechanisms (8) and in that study, morphine increased serum NO concentration in 2K-1C animals which approached normal level. Thus, it seems that the increased serum NO level could not completely explain enhanced angiogenesis in morphine-treated hypertensive animals. On the other hand, there is a relationship between NO and angiotensin II (Ang II). It was shown that high Ang II level decreases NO bioavailability by promoting oxidative stress (9). Thus, morphine probably reduced plasma renin activity and Ang II levels which results in increased NO level. More studies need to clarify the exact role and mechanism of angiogenesis during morphine administration.
